# Annotations

**Published:** 1894-01-20

**Authors:** 


### ANNOTATIONS.

### Some Dangers of Self-drugging.

THAT extremely useful committee of the British Medical Association, the Therapeutic Committee, has just issued its special report of an inquiry instituted to bring out and define the dangers said to be associated with certain drugs largely used by the members of the public on their own initiative. The chief of these drugs is antipyrin, one of the favourite remedies of the modern victims of neuralgia. The investigation has formally established the fact that in medical hands antipyrin is often an excellent remedy for neuralgic pains; it has also proved definitely that the following results have sometimes ensued upon the use of the drug, even in skilled hands; and that therefore those non-medical persons who use it on their own responsibility incur risks and dangers of an exceedingly serious kind. The dangerous symptoms noted after the administration of ordinary doses have been, among others, "alarming depression, excessive sweating and partial collapse, vasomotor pains and lividity, a condition of mania, dizziness and loss of power in the legs, alarming faintness, loss of speech lasting twenty-four hours, cardiac weakness and irregularity, cyanosis, dyspnœa with much nervous excitement, collapse and death." This is a considerable list of dangerous results from the use of one single drug, but it is by no means exhaustive. Those who have been in the habit of taking antipyrin without submitting themselves to the watchful care of the skilled medical attendant, should certainly now hold their hands. Even medical men, after the publication of this report, will feel that the drug is an edged tool of a decidedly dangerous character, and must be employed with a caution hitherto considered unnecessary.

### Albuminuria and Life Assurance.

NOTWITHSTANDING the more intelligent comprehension of the varying significance of albuminuria by the critical members of the medical profession, it is still the case in common practice that the presence of albumin is regarded as pathognomonic of Bright's disease, and that most persons in whom this symptom is detected are rejected by life assurance offices. There can be little doubt that much excellent business is thus lost to the offices, and that many persons are prevented from making provision for their families in this way who ought not to be prevented. The kidney, it must be remembered, has a wider range of eliminatory function than any other organ in the body. So far as appears it will eliminate almost anything which may happen to be present in the blood. That being so the wonder is, not that healthy persons sometimes show the symptom of albuminuria, but that they do not show it ten times oftener than they do. Some facts and deductions may be of service to the busy practitioner who has not time to be carefully critical. For example, the morning urine of healthy persons is the least liable to be albuminous; the urine passed later, and especially after fatiguing labour, is certain to be albuminous in a considerable proportion of healthy persons. Experiments made on a large number of healthy soldiers showed that only 4·2 per cent. had albumin in their morning urine, but 16 per cent. in that passed at midday. Of course, with the ordinary practitioner, the difficulty is to establish a working rule whereby to separate the healthy from the diseased "albuminuriacs," to coin a word. Dr. Saundby lays down a rule which, when applied by experienced persons with reasonable discrimination, will be likely to give generally safe results. The rule is this, that an "albuminuriac" may, other things being equal, be accepted for life assurance if "the urine is normal in all other respects; if there are no tube casts, if the amount of solid matter excreted is sufficient, if there are no signs of cardiac hypertrophy or of high arterial tension, and if there are no retinal changes and no œdema." To this one would add that several examinations should be made, and if the albuminuria should prove to be only occasional, a very reassuring point would be established. Most of these points can be easily cleared up by the ordinary practitioner who examines conscientiously. The retinal examination may sometimes demand the services of a specialist. But this both the ordinary examiner and the office should cheerfully consent to, in the interests alike of the office and the examinee. The classification of all "albuminuriacs" under the one general head of Bright's disease cases is somewhat of a reproach to modern medical science and art.

### Should Midwives be Registered?

A SOCIETY—by no means the first—is being established among medical men to promote the registration of midwives. Ought medical men to support the objects which the society has in view? And if they ought, what are the grounds which constitute the obligation? It is desirable that every medical man who decides to support the society should clearly understand the grounds of his action, because it is certain that he will be assailed by his brethren, who fear that midwives registration means injustice to registered medical men. For our part we do not think medical men will be injured, but benefited by the registration of midwives. What, in brief, are the reasons which induce medical men of the thoughtful and disinterested class to encourage the registration of midwives? Most of those reasons are set forth in the "rules" of the Midwives Registration Association. Among the "objects" aimed at by the Association are first, the education and supervision of midwives; secondly, that the education and supervision shall be conducted by legally qualified medical men; and thirdly, that the legally qualified medical men shall be representative of every department of the profession. Such objects we cannot but consider entirely worthy. In these democratic times the medical profession is really the State, so far as the health and lives of the people are concerned. What we should set before ourselves as a collective profession is the "greatest health of the greatest number." In order to secure this we must insist that everybody who is allowed by law or custom to practice the medical art, or any branch of it, shall be competently educated and adequately supervised. Any lower aims than these we hold to be absolutely unworthy of an educated class, and not fit even to be named in a profession which boasts itself as above all things "humane."

